# Entomophagy practices, use patterns, and factors influencing perception and consumption frequency of edible insects in the Republic of Benin

**DOI:** 10.1186/s13002-023-00626-z

**Published:** 2023-11-22

**Authors:** Corinne Mèdéou Anagonou, Laura Estelle Yêyinou Loko, Anicet Gbeblonoudo Dassou, Joelle Toffa, Innocent Djegbe, Manzid Saliou, Alexandre Dansi

**Affiliations:** 1 Laboratory of Applied Zoology and Plant Health (ZASVE), National High School of Applied Biosciences and Biotechnologies (ENSBBA), National University of Sciences, Technologies, Engineering and Mathematics (UNSTIM), PO Box 14, Dassa-Zoumé, Benin; 2 Laboratory of Biotechnology, Genetic Resources and Plant and Animal Breeding (BIORAVE), ENSBBA, UNSTIM, PO Box 143, Dassa-Zoumé, Benin; 3 Département des Sciences de la Vie et de la Terre, Ecole Normale Supérieure de Natitingou, UNSTIM, Natitingou, Bénin; 4 Laboratory of Biomathematics and Forest Estimates (LBEF), University of Abomey-Calavi (UAC), Abomey-Calavi, Benin

**Keywords:** Entomophagy, Consumers’ preferences, Consumption frequency, Influencing factors, Entomotherapy

## Abstract

**Background:**

Edible insects are important sources of essential nutrients and have the potential to contribute to malnutrition reduction and food security in the Republic of Benin. However, their consumption is always restricted to a limited number of sociocultural groups. To determine how the consumption of insects could be promoted as an alternative food source, this study documents the endogenous knowledge associated with edible insects and, the main factors that govern their perception and frequency consumption.

**Methods:**

A survey was conducted towards 479 rural households consuming edible insects through 91 villages of Atacora, Alibori, Zou, and Plateau departments using individual interviews with a semi-structured questionnaire. The survey was focused on the inventory of edible insects and the documentation of consumers’ acceptance, frequencies and motive reason of consumption, local uses, and accessibility to edible insects. Samples of edible insects were collected and preserved in 70% alcohol for taxonomic identification.

**Results:**

The majority of surveyed people (79.1%) were consumers of edible insects since many years ago (29.1 ± 17.2 years). Insect species belonging to 17 genera of 7 families and 3 orders of insects were used as food, with *Brachytrupes membranaceus* Drury being the most widespread and consumed. Six factors affecting edible insect availability were identified with the chemical pollution as the most important. Besides their food use (63.2%), edible insects in the study area were used for several purposes. We find that ethnicity, religion, age, education level, and monthly frequency of insect consumption are the main factors influencing the local perception of edible insects. Indeed ethnic group, religion ethnicity, and market accessibility have a positive influence on edible insect consumption frequency. The Hierarchical Clustering of Principal Components has allowed us to classify the interviewees into 3 groups with different perceptions of entomophagy and their characteristics will make it possible to better orient the strategies for promoting entomophagy in the Republic of Benin.

**Conclusions:**

Religion and tradition are among the main factors that influence entomophagy in Benin Republic. The development of a national strategy to promote entomophagy should take into account the recorded insect consumption motivations, and their different uses by each ethnic group, and mainly target young people.

## Background

Consumed for centuries, edible insects were first promoted as an alternative sustainable source of nutrients for humans and livestock by Meyer-Rochow [1] who recommended that WHO and FAO support the idea. After the report published by the FAO on edible insects [2], some authors supported the assumption that edible insects could provide an alternative source of protein to meet the demand of rapid population growth [3, 4]. Indeed edible insects have been reported to be as rich in proteins, fats, and minerals as traditional meats [1, 5]. Some edible insect species contain more protein than some meat [6], more iron than spinach, as much vitamin B12 as salmon, amino acids, as well as high calcium, Omega 3, and fibre contents [5, 7]. The low environmental pollution, low water usage and land, and fast returns on investment are other benefits of edible insect’s production [8, 9]. Unfortunately, despite all these advantages, edible insects’ acceptability and consumption remain today the biggest challenge for humans and industry [10]. Indeed, several factors from socio–demographic, psychological (insect phobia, feelings of disgust), and cultural influence consumer attitudes and behaviour to accept insects as food and feed [11].

In the Republic of Benin, consumption of edible insects is restricted to a limited number of sociocultural groups, such as the Waama, Dendi, Ottamari, Bariba and Nagot. Although some studies have been conducted on edible insects in Benin, they are mainly limited to their diversity, gathering method, collection place, seasonal availability, consumption patterns, and consumer perception [6, 12–16]. However, little is known about the consumption frequency of edible insects, their variation across different consumption zones in Benin, their traditional uses by Beninese people, and the factors that influence their accessibility. While this information is important not just for the preservation of traditional knowledge associated with edible insects but also for their conservation and sustainable uses.

The development of strategies allowing the acceptance of entomophagy by Beninese populations requires a better understanding of the factors influencing the perception and consumption of edible insects. Indeed, several factors such as consumers’ socio-demographic characteristics, consumers’ external environment, and factors related to the insects consumed are known as influencing the acceptance or rejection of entomophagy [17]. Unfortunately, very few studies have focused on the factors influencing the acceptance and practice of entomophagy by Beninese people. Therefore, no policy is implemented to promote entomophagy despite its potential to reduce malnutrition and food insecurity. Indeed, edible insects, due to their richness in proteins and essential nutrients [18] offer an opportunity to reduce the rate of malnourished children and food insecurity in Benin. Furthermore, to our knowledge, no study has collected data both in the north and in the south of Benin in order to have a general view of the entomophagy practices that are crucial for the implementation of a national policy for the promotion of edible insects. In order to encourage entomophagy in Benin and directly contribute to the attainment of the Sustainable Development Goals (SDGs), it is necessary to carry out investigations on indigenous knowledge related to edible insects and determine factors that promote or hinder the expansion of entomophagy.

The aim of this study was to (1) document the consumption frequency, and local uses of edible insects; (2) examine the local perception of consumers about entomophagy; (3) investigate factors influencing consumers’ perception and consumption of edible insects.

## Methodology

### Study area

**Fig. 1:**
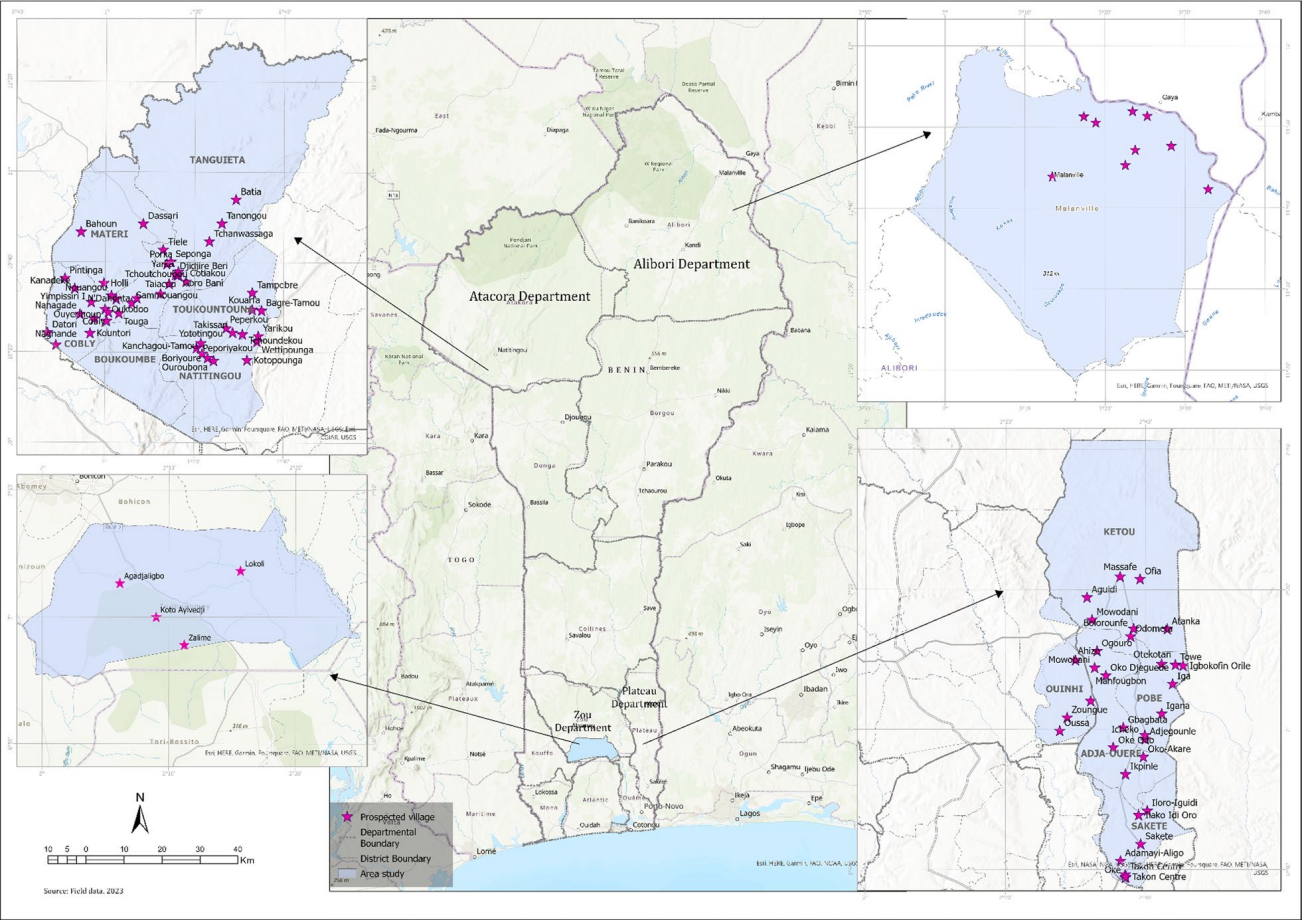
Map of the Republic of Benin showing the localisation of the surveyed villages

The survey was carried out in 91 villages located across the departments of Atacora (Latitudes/ Longitude: 10° 45′ 0" N/1° 40′ 0" E), Alibori (Latitudes/ Longitude: 11°19′ 0" N/2°55′ 0" E), Zou (Latitudes/ Longitude: 7° 15′ 0" N/2° 10′ 0" E) and Plateau (Latitudes/ Longitude: 7° 10′ 0" N/2° 34′ 60" E) in the Republic of Benin (Fig. 1). These departments are known as the main areas of consumption of edible insects in Benin [6, 12–14, 16]. The departments of Atacora and Alibori located in the north of Benin are characterized by a Sudano-Sahelian climate (humid tropical) with a unimodal rainfall regime with a rainy season (April to October) and a dry season (November to March). The average annual rainfall varies from 700 to 1,200 mm with average annual temperatures varying between 26 and 27 °C. This region has a diversity of soils (raw mineral soils, cambisols, tropical ferruginous soils, ferralitic soils, and hydromorphic soils) with woody, shrubby, and grassy vegetation. The departments of Zou and Plateau located in south Benin have a bimodal climate (Sudano-Guinean type) with two rainy seasons and two dry seasons A major rainy season (March to July), a minor dry season in August, a minor rainy season (September to November) and a major dry season (December to March). Annual rainfall varies from 900 to 1300 mm with dominant soils of ferralitic type. The average annual temperature is 28 °C. In the study area, agricultural activities are most dominant, and indigenous knowledge is extensive in terms of the uses of plants and animals for medicinal and cultural purposes [12].

### Sample size

The sample size for the study was determined using the formula of Dagnelie [19]:

$$n = \frac{{{\text{Z}}^{2} \times p\left( {1 - p} \right) \times N}}{{Z^{2} \times p\left( {1 - p} \right) + \left( {N - 1} \right) \times ET^{2} }}$$

"n" the required sample size."N" the actual size of the population."ET" the standard error (margin of error) tolerable for the survey: ET = 0.05."z" the normal standard value of the confidence interval: Z ≈ 1.96."p" proportion of rural population in study areas.

The size n of the sample is thus substantially equal to 400 individuals. The number of people interviewed per locality was determined by proportionality considering the size of the population of each locality. However, only people who had eaten edible insects at least once in their life were included in the survey.

### Selection of respondents

The respondents of this study were people who had practiced entomophagy in the past and who, today, continue or not to consume edible insects. These respondents were chosen from the villages of the Alibori, Atacora, Zou and Plateau departments, which represent the major regions of consumption of edible insects in the Republic of Benin [6, 12–14, 16]. In each department, the surveyed villages were chosen on the basis of previous studies mentioning edible insects carried out in the Republic of Benin [12–14, 16]. Within each selected village, the surveyed households practicing entomophagy were chosen with the help of the village chief, and using the snowball method [20]. In total, 479 rural households consuming edible insects were surveyed in the study area (Table 1).

**Table 1:** Socio-demographic characteristics of respondents by surveyed department

Variables	Levels	Alibori N = 90	Atacora N = 228	Plateau N = 110	Zou N = 51	Total N = 479	Percentage
*Gender*							
	Female	39	78	46	16	179	37.4
	Male	51	150	64	35	300	62.6
*Age groups*							
Female	Young (age < 30)	14	6	13	3	36	20.2
	Adult (age between 30 and 60)	22	62	26	13	123	68.7
	Older person (age ≥ 60)	3	10	7	–	20	11.2
Male	Young (age < 30)	24	17	14	6	61	20.3
	Adult (age between 30 and 60)	23	120	45	29	217	72.3
	Older person (age ≥ 60)	4	13	5	–	22	7.3
*Marital status*							
	Married monogamous	48	130	50	17	245	51.1
	Married Polygamous	22	58	39	20	139	29
	Single	20	40	21	14	95	19.9
*Sociolinguistic groups*							
	Ottamari	–	181	–	–	181	37.8
	Yorouba	1	1	107	25	134	28
	Dendi	86	4	–	–	90	18.8
	Fon	3	3	1	26	33	6.9
	Cotimba	–	33	–	–	33	6.9
	Adja	–	1	2	–	3	0.6
	Bariba	–	3	–	–	3	0.6
	Peulh	–	1	–	–	1	0.2
	Yom-Lokpa	–	1	–	–	1	0.2
*Religion*							
	Christian	–	116	77	28	221	46.1
	Muslim	90	28	16	7	141	29.5
	Animist	–	84	17	16	117	24.4
*Highest level of education*							
Female	Illiterate	28	46	35	12	121	67.6
	Primary	7	13	8	2	30	16.8
	Secondary	4	17	3	2	26	14.5
	University	–	2	–	–	2	1.1
Male	Illiterate	25	61	39	22	147	49
	Primary	5	37	16	10	68	22.7
	Secondary	18	42	8	3	71	23.7
	University	3	10	1	–	14	4.7
*Main sources of income for the family*							
	Agriculture	33	102	52	44	231	48.2
	Trade	43	56	33	5	137	28.6
	Crafts	14	64	22	1	101	21.1
	Employee	–	4	3	1	8	1.7
	None	–	2	–	–	2	0.4

### Survey

Data were collected during face-to-face interviews on the basis of a pre-tested and revised questionnaire. The survey was carried out during the rainy season (July to October 2022) by two trained investigators. The oral consent of the participants was taken after the presentation of the objectives of the survey. Translators were recruited locally and impregnated with the questionnaire to facilitate exchanges with the interviewees. The number of surveyed households varied from one department to another, depending on the edible insect’s consumers at the level of each surveyed village (Table 1). The collected data focused primarily on the socio-demographic characteristics of respondents (gender, age, ethnicity, religion, type of household, diet, income source, and education level), which are known as having a positive impact on respondents' willingness, perception, consumption, and acceptance of edible insects [21–24]. Among the respondents, 62.63% were men and the majority were illiterate (55.95%). The great majority of interviewees were married (80.17%) with a predominance of monogamous households (51.15%). Ottamari (37.79%), Yoruba (27.97%), and Dendi (18.8%) were the most represented ethnic groups. The respondents were Christians (45.93%), Muslims (29.44%), or Animists (24.63%). Agriculture was the main source of income (48.23%) of the surveyed households (Table 1). The second part of the survey was focused on the inventory of edible insects as well as the documentation of frequency of consumption, parts consumed, consumer preferences, seasonal availability, environmental factors affecting the diversity of edible insects, and traditional uses. The perception of the surveyed households on the edible insect’s consumption was assessed through a 5-point Likert scale (1 = completely disagree, 5 = completely agree) [9]. Samples of edible insects were also collected and stored in plastic boxes containing 70° ethanol for identification in the laboratory. Voucher samples were deposited at the Laboratory of applied entomology (LEnA) of the National High school of Applied Biosciences and Biotechnologies (ENSBBA).

### Data analysis

The descriptive analysis was carried out to summarize the sociodemographic characteristics of the surveyed households. Chi-square (χ^2^) or Fisher test was performed to assess the relationship between insect consumption practices and ethnic groups. Principal component analysis (PCA) using the Facto Mine R package was performed [25] to describe the different uses of edible insects and respondents' perceptions of insect consumption with respect to ethnic groups.

The use value (UV) of each of the edible insect species inventoried in the study area was calculated following Alves et al. [26] using the formula:

$${\text{UV}} = \sum {\frac{{{\text{Ni}}}}{{{\text{Nt}}}}}$$

where Ni is the number of respondents who mentioned the use of the edible insect species, and Nt is the total number of respondents.

The fidelity level (FL), the percentage of respondents claiming the use of a certain species of edible insects for the same major purpose, was calculated for the most frequently reported diseases or ailments as:

$${\text{FL }}\left( \% \right) \, = \, \left( {{\text{Np }}/{\text{ N}}} \right) \, \times \, 100$$

where Np is the number of respondents that claim of use an edible insect’s species to treat a particular disease, and N is the number of respondents that use the edible insects as a medicine to treat any given disease [27].

Hierarchical Clustering on Principal Components (HCPC) was performed to assess the influence of social, cultural, and economic characteristics of respondents on the perception of insect consumption. The HCPC consisted of a Multiple Correspondence Analysis (MCA) realised on the perception measures and on the social characteristics of the respondents, followed by a hierarchical clustering analysis (HCA) on the principal components from the MCA. To select the variables for each perception group, we measured the difference between the class values and the overall values. These statistics were converted into a criterion called the value test (V-test) to perform a selection on the variables, and thus determine the most characteristic variables [28, 29]. The most characteristic variables of a group were those for which the test of associated values was greater in absolute value than 2. Moreover, if this test of value was positive for a variable, it had a high value in the class considered. On the other hand, if the value was negative, the variable had a low value for the class.

A multivariable logistic regression of the outcome of edible insect consumption (No/Yes) on the independent variables (age group, gender, ethnic group, religion, education level, main source of income, and market accessibility) was conducted to determine their association. The conditional association of the different independent variables with the outcome was quantified using odds ratios.

All the analyses were performed in R version 4.3 [30] and a significance level of 5% was considered. The graphs for the descriptive analysis were constructed using the package ggplot2.

## Results

### Consumption patterns of edible insects

**Fig. 2:**
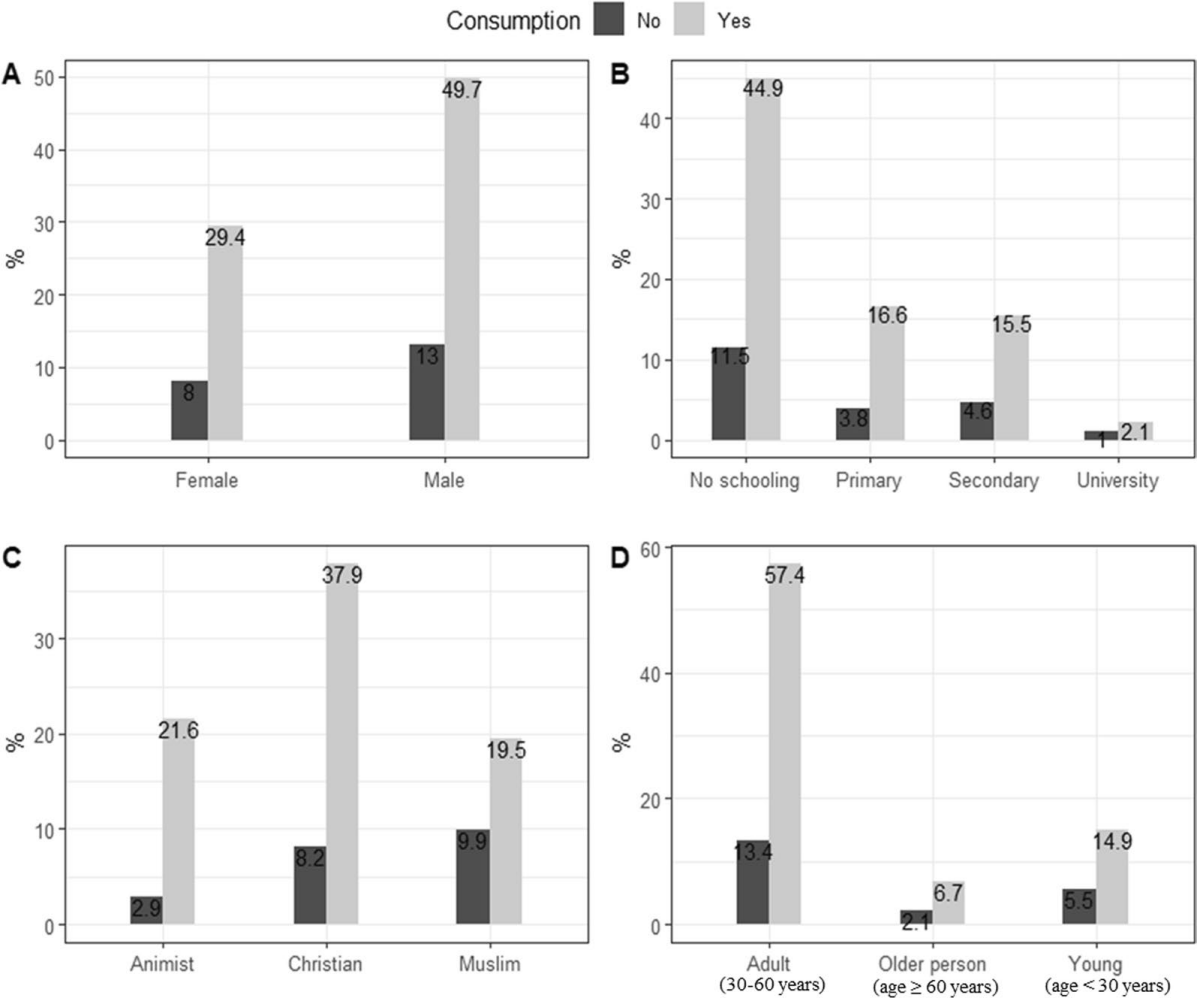
Variation in consumption of edible insects depending on **A** gender, **B** educational level, **C** religion and **D** age in the study area

The majority of the surveyed people (79.1%) were still consumers of edible insects and since several years ago (29.1 ± 17.2 years). The remaining surveyed people (20.9%) have consumed edible insects at least once in the past but no longer consume them. Most of the surveyed men (49.7%) consume edible insects against 29.4% of the surveyed women. Contrary to the other departments where the consumption of insects by the surveyed people is more recent, those surveyed in the Atacora department have consumed them since their childhood (39.3 ± 14.3 years of consumption on average). Among the nutrient’s sources of the surveyed households, edible insects represent 10 and 30% of annual intake (Table 2). About 33.6% of the surveyed people consumed edible insects at least once a month and only 21.3% consume them almost daily. There were no significant differences according to gender (*p* = 0.795) and level of education (*p* = 0.402) in the frequency of consumption of edible insects. Throughout the study area, insect consumption is usually family-based (79.3%). However, in the plateau department, entomophagy is much more practiced by parents (Table 2). Fried insects (71.8% of the surveyed people) are the preferred form of consumption in the study area. A difference in terms of the frequency of insect consumption was observed across departments (*p* < 0.001), age (*p* =  < 0.001), and religion (*p* =  < 0.001). More illiterate (44.9%), Christian (37.9%), and adult (57.4%) respondents continue to practice entomophagy compared to others (Fig. 2).

**Table 2:** Consumption frequency and use patterns of edible insects in the study area

Variables	Alibori (*N* = 90)	Atacora (*N* = 228)	Plateau (*N* = 110)	Zou (*N* = 51)	Total (*N* = 479)	Percentage
Consumption						
Respondents practicing entomophagy	55	204	79	41	379	79.1
Number of years of insect consumption						
[0–30]	89	60	71	47	267	55.7
[31–60]	1	151	39	4	195	40.7
[61–70]	–	17	–	–	17	3.6
Proportion as source of nutrients (%)						
10	79	169	100	35	383	80
20	6	54	10	10	80	16.7
30	5	5	–	6	16	3.3
Frequency of consumption						
Once per day	16	72	9	5	102	21.3
Once per week	16	69	35	14	134	28
Once per month	28	73	37	23	161	33.6
Once per year	30	14	29	9	82	17.1
Family consumption						
Parents only	1	13	44	4	62	12.9
Children only	10	10	7	2	29	6.1
Old person only	1	5	2	–	8	1.7
All family members	78	200	57	45	380	79.3
Mode of consumption						
Raw	–	5	–	–	5	1
Boiled	5	5	1	7	18	3.8
Smoked	7	47	23	21	98	20.5
Fried	75	166	85	18	344	71.8
Dried	3	5	1	5	14	3
Uses						
Only for food	85	99	68	51	303	63.2
Traditional medicine	–	68	23	–	91	19
Spiritual uses	–	26	16	–	42	8.8
Poultry feed	–	35	3	–	29	6.1
Animal trap	–	6	–	–	6	1.3
Sell	5	–	–	–	5	1
Fishing	–	–	3	–	3	0.6

### Motivation for insect consumption

**Fig. 3:**
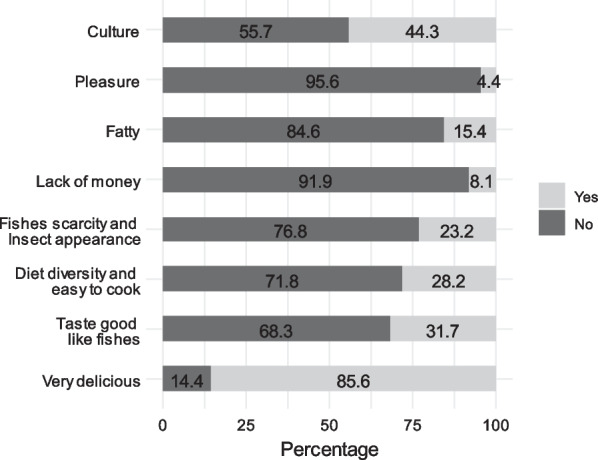
Incentives for insect consumption in the study area

Eight reasons were mentioned by the surveyed people for insect consumption (Fig. 3). The delicious taste of edible insects (85.6%) and the cultural values (44.3%) associated with them were mentioned as the most important reasons. The good taste similar to fish (31.7%), the seasonal fish scarcity (23.2%), the food diversification, and the ease of preparing edible insects (28.2%) were listed by some households as reasons for insect consumption. While, some surveyed households consume edible insects specifically for their fatty content (15.5%), by pleasure (8.1%), or by lack of money (4.4%).

### Diversity of edible insects

**Fig. 4:**
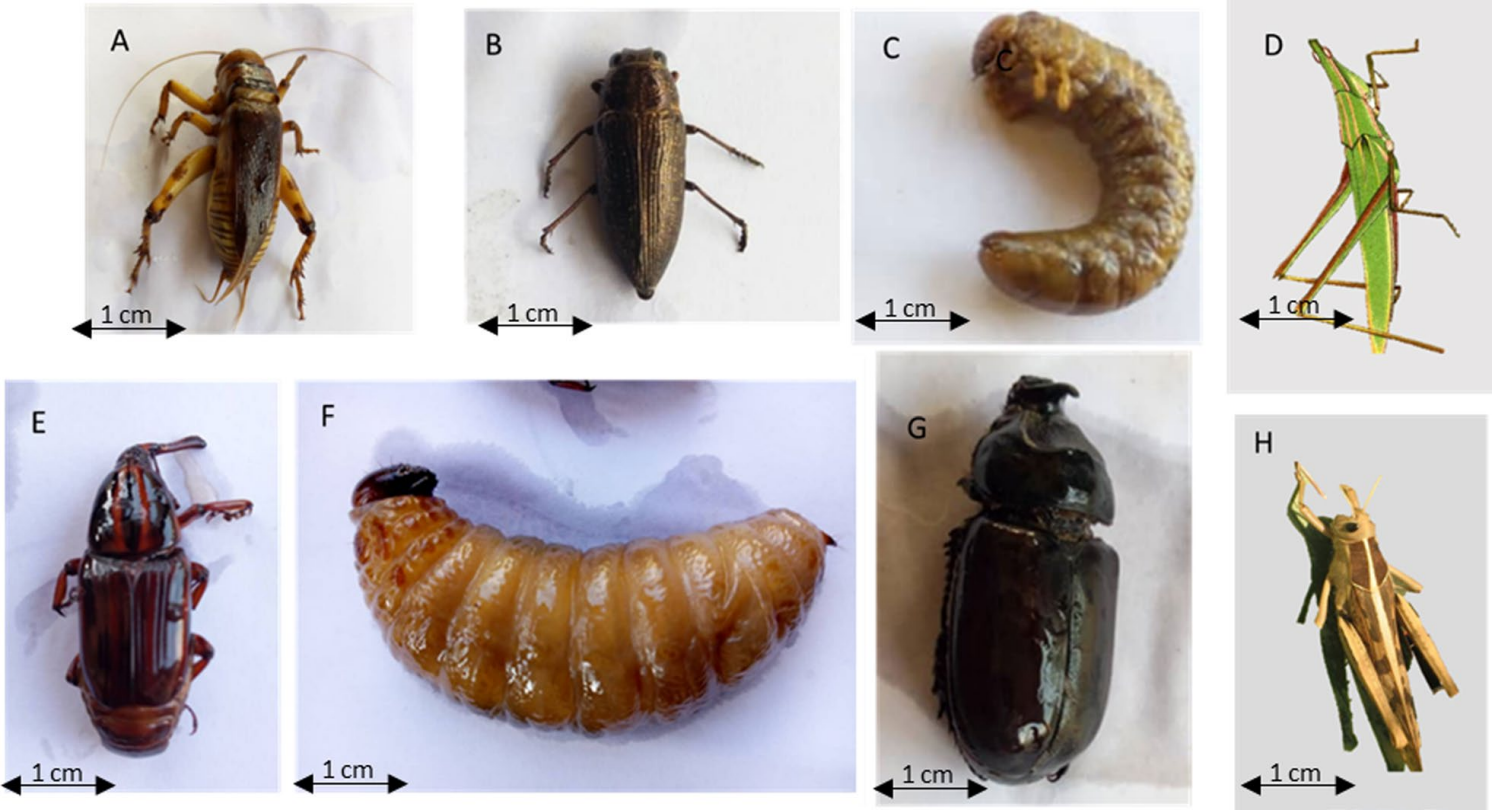
Pictures of some edible insects collected in Southern Benin. Scale: 1:1. **A** *Brachytrupes membranaceus*; **B** *Lampetis gorilla (Thomson)*; **C** *Lava of Oryctes ohausi Minck*; **D** *Truxalis sp*; **E** *Adult of Rhynchophorus phoenicis Fabricius*; **F** *Lava of Rhynchophorus phoenicis, Fabricius*; **G** *Adult of Oryctes ohausi Minck*; **H** *Ornithacris turbida cavroisi*

**Fig. 5:**
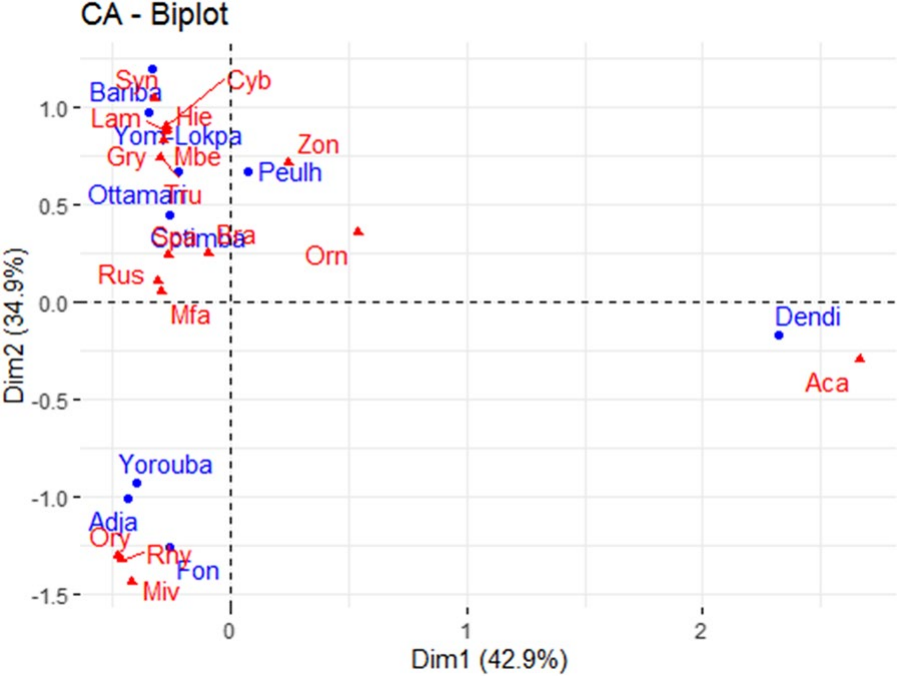
Distribution of edible insects according to the surveyed sociolinguistic groups. Zon: *Zonocerus variegatus,* Tru: *Truxalis* spp, Cyb: *Cybister* sp., Lam: *Lampetis gorilla,* Rus: *Ruspolia differens,* Miv: *Macrotermes ivorensis,* Gry: *Gryllus bimaculatus,* Bra: *Brachytrupes membranaceus,* Msu: *Macrotermes subhyalinus,* Spa: *Spathosternum pygmaeum,* Mbe: *Macrotermes bellicosus,* Aca: *Acanthacris ruficornis citrina,* Orn: *Ornithacris turbida cavroisi,* Ory: *Oryctes monoceros,* Hie: *Hieroglyphus africanus,* Mfa: *Macrotermes falciger,* Rhy:* Rhynchophorus phoenicis*

Insect species belonging to 17 genera were used as food and that some were identified to species level in the study area (Table 3). The inventoried insects belong to 7 scientific families with the order of the Orthoptera (52.9%) being the most represented. Fourteen, 7 and 6 edible insect species were listed respectively in the departments of Atacora, Plateau, and Zou. While, only 3 edible insect species were listed in the Alibori department. In the study area, the most consumed insects were *Brachytrupes membranaceus* Drury (Fig. 4), followed by *Macrotermes subhyalinus* Rambur, *Macrotermes bellicosus* Smeathman, and *Rhynchophorus phoenicis* Fabricus (Table 3). However, only, *B. membranaceus* was consumed by people in all the surveyed departments. The insect species consumed by the surveyed people varied according to ethnic groups (Fig. 5). *Oryctes monoceros* Olivier, *R. phoenicis*, and *Macrotermes ivorensis* Grassé & Noirot were more consumed by Yoruba, Fon et Adja ethnic groups. While *Acanthacris ruficornis citrina* Serville was more consumed by the Dendi ethnic group. The thirteen remaining species were consumed mainly by the other ethnic groups in the study area (Fig. 5).

**Table 3:** Insect species consumed by respondents in the study area

Order	Family	Voucher number	Scientific names	Common name	Local name (ethnic group)	Seasonal availability	Form of consumption	Departments				Nc	Nu	UV
								At	Al	Z	Pl			
Blattoptera	Termitidae	129	*Macrotermes bellicosus* (Smeathman)	Termites	Iriiri (Ottamari), Tchillili (Yom-Lokpa)	Mar–Nov	Raw, boiled, roasted, fried	+	−	−	−	108	5	0.71
		130	*Macrotermes subhyalinus* (Rambur)	Termites	Iba (Yorouba), n'same (Ottamari), Cafléko (Fon)	Jan–Dec	Boiled, roasted, fried, dried	−	+	+	+	174	6	0.85
		131	*Macrotermes ivorensis* (Grassé & Noirot)	Termite	Etoutou (Yorouba), Toutou (Fon)	May–Nov	Raw, roasted, fried, dried	−	−	+	+	32	3	0.42
		132	*Macrotermes falciger* (Gerstäcker)	Termite	Toubou (Bariba), Gnommi (Yom-Lokpa)	Mar–Dec	Roasted, fried	+	−	+	−	3	3	0.42
Orthoptera	Acrididae	136	*Truxalis* spp.	Short horned grasshoppers	Chaubafranca (Ottamari)	Aug–Dec	Boiled, roasted, fried, dried	+	−	−	−	44	4	0.57
		140	*Spathosternum pygmaeum* (Karsch)	Grasshoppers	Igbe (Yorouba,), Kanankoun (Ottamari), Tchaganlikou (Cotimba)	Sept–Dec	Boiled, roasted, fried, dried	+	−	−	+	33	6	0.85
		137	*Ornithacris turbida cavroisi* (Finot)	Cricket	Mantchoubou (Ottamari, Dendi)	Sept–Jan	Roasted, fried	+	+	−	−	27	5	0.71
		141	*Gryllus bimaculatus* (De Geer)	Two-spotted cricket	Gboo (Bariba), Tabante (Ottamari)	Aug–Dec	Roasted, fried, dried	+	−	−	−	84	5	0.71
		138	*Hieroglyphus africanus* (Uvarov)	Rice grasshopper	Sosore (Ottamari)	Aug–Dec	Roasted, fried	+	−	−	+	4	3	0.42
		139	*Acanthacris ruficornis citrina* (Serville)	Garden locust	Manchougou (Ottamari)	Sept–Dec	Raw, roasted, fried, dried	+	−	−	+	81	2	0.28
	Gryllidae	133	*Ruspolia differens* (Serville)	Longhorn grasshoppers	Don (Dendi), Dow (Bariba), Dounadoudi (Cotimba)	Apr–Dec	Roasted, fried	+	−	−	−	4	3	0.42
		134	*Brachytrupes membranaceus* (Drury)	Tobacco cricket	Hyre (Yorouba), Tambaga (Ottamari), Baadra (Cotimba), Botaclé (Fon)	Sept–Dec	Raw, boiled, roasted, fried, dried	+	+	+	+	161	7	1
	Pyrgomorphidae	135	*Zonocerus variegatus* (Linné)	Variegated grasshopper	Alantakare (Ottamari)	Sept–Dec	Roasted, fried	+	−	−	−	12	4	0.57
Coleoptera	Buprestidae	128	*Lampetis gorilla* (Dejean)	–	Dinantchacane (Ottamari)	Jun–Oct	Roasted, fried	+	−	−	−	26	4	0.57
	Dytiscidae	127	*Cybister* sp.	Water beetle	Cotondousre (Ottamari)	Jan–May	Roasted, fried	+	−	−	−	3	1	0.14
	Dynastidae	126	*Oryctes monoceros* (Olivier)	Coconut beetle	Ogongo (Yorouba)	Apr–Dec	Roasted, fried	−	−	+	+	17	3	0.42
	Curculionidae	125	*Rhynchophorus phoenicis* (Fabricius)	African Palm Weevil	Woiwo (Yorouba), Dekpomintonvi (Fon)	Mar–Dec	Raw, boiled, roasted, fried, dried	+	−	+	−	119	4	0.57

*At* Atacora department, *Al* Alibori department, *Zo* Zou department, *Pl* Plateau department; *Nc* Number of citations, *Nu* Number of uses, *UV* Use value

### Factors influencing the accessibility to edible insects

**Fig. 6:**
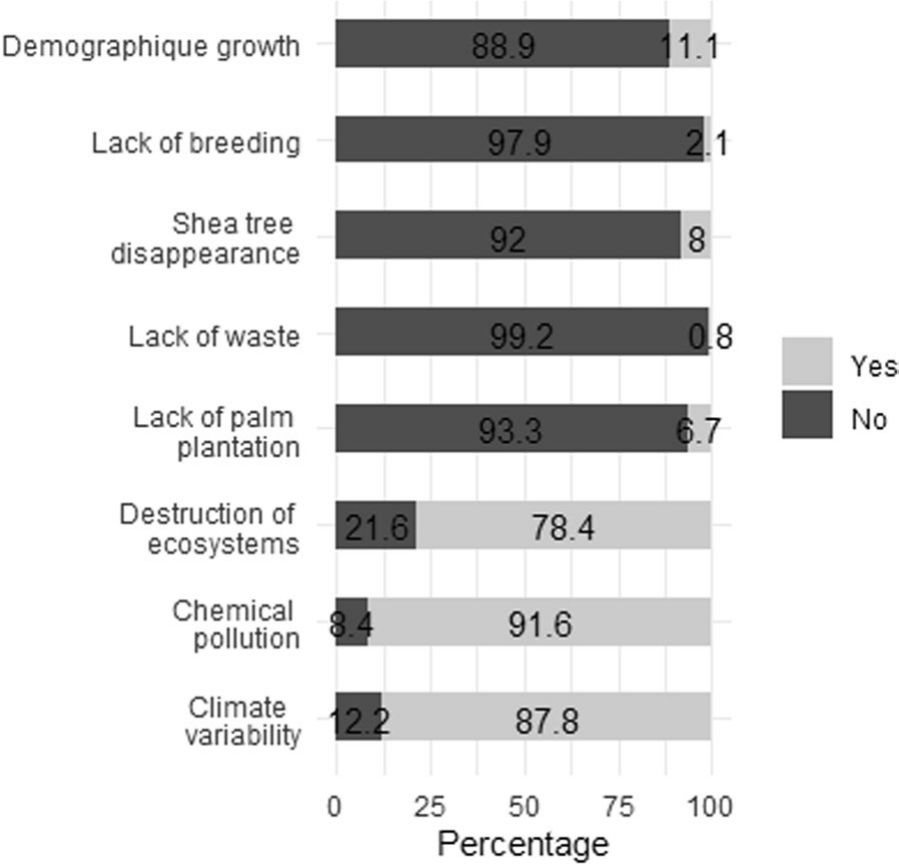
Factors affecting the accessibility to edible insects in the study area

The collection of edible insects was done mainly in the wild with a period of accessibility coinciding mainly with the rainy season. Edible insects are mainly collected from crop fields (38.3%), houses (37.3%), savannas (15.2%), forests (3.4%), public places (2.9%), and mountains (2.9%). Six factors affecting the accessibility to edible insects were identified in the study area (Fig. 6). The most important factors were chemical pollution in edible insect collection fields (91.6% of surveyed people), climate variability (87.8%), and destruction of ecosystems (78.4%). Twenty-five chemical pesticides were perceived by the surveyed people as contributing to the chemical pollution of edible insect collection fields. Among them, 10 herbicides, 13 insecticides, and 2 fungicides were recorded (Table 4). The most used herbicides in edible insect collection fields are Nicomais 40 SC (12.1% of responses) and Kalach Extra 70 SG (9.8%) while Sunpyrifos 48% EC (12.7%) was the most cited insecticide.

**Table 4:** Pesticides used in crop fields where edible insects are collected by the surveyed consumer people

Main use^c^	Commercial name	Active ingredient	Chemical class^a^	Toxicological class^b^	Department				Study area (%)
					Alibori (N = 2)	Atacora (N = 76)	Plateau (N = 41)	Zou (N = 46)	
Insecticide	Sunpyrifos 48% EC	Chloropyriphos-Ethyl (480 g/l)	OP	II	–	21	16	2	12.7
	Cotonix 328 EC	Deltamethrin (12 g/L) + Chlorpyriphos-Ethyl (300 g/L) + Acetamiprid (16 g/L)	Pyr + OP + Neo	III	–	23	3	2	9.1
	Shark	Deltamethrin1% + trizophos 35% EC	Pyr + OP	II	–	8	5	6	6.2
	Lambdacal P 214 EC	Lambda-Cyhalothrin 12 g/l + Profenofos 200 g/l	Pyr + OP	II	–	–	–	12	3.9
	Pacha 25 EC	Lambda-Cyhalothrin 15 g/l + Acetamiprid 10 g/l	Pyr + Neo	II	–	–	–	9	2.9
	BaseLine	Bifenthrin 23.4%	Pyr	II	–	6	–	–	1.9
	Manco 80 WP	Mancozeb 800 g/kg	DTCs	U	–	–	–	3	0.9
	Thalis 56 EC	Emamectin Benzoate 24 g/l + Acetamiprid 32 g/l	Neo + Ave	II	–	–	–	2	0.7
	Thunder 145 Q-TFQ	Beta-cyfluthrin (45 g/L) + Imidacloprid (100 g/L)	Pyr + Neo	Ib + II	–	–	2	–	0.7
	Cypercal 50 EC	Cypermethrin 50 g/L	Pyr	II	–	–	–	1	0.3
	TopBio	Azadirachtin	Azadirachtins	U	–	–	–	1	0.3
	Pirpro	Pyriproxyfen 10 g/l	Pyr	III	–	2	–	2	1.3
Insecticide/Acaricide	Acarius 018 EC	Abamectine 18 g/l	Ave	Ib	–	–	–	14	4.6
Herbicide	Nicomais 40 SC	Nicosulfuron 40 g/l	Pyr	III	–	–	1	36	12.1
	Kalach Extra 70 SG	Glyphosate 700 g/l	OP	III	2	27		1	9.8
	Herbextra 720 SL	2.4- Sel de D diméthylamine 720 g/l	Ary	II	–	6	17	5	9.1
	Glycel 710 SG	Glyphosate 710 g/kg	OP	II	–	9	8	–	5.6
	Slasher weedkiller	Nonanoic acid 525 g/l	OP	III	–	–	12	5	5.6
	Adwuma Wura 480 SL	Glyphosate 480 g/l	OP	III	–	7	8	–	4.9
	Atrazine 80 WP	Atrazine 800 g/L	T	III	–	–	–	12	3.9
	Killer 480 SL	Glyphosate 480 g/l	OP	III	–	6	–	–	1.9
	Finish 68 SG	Glyphosate 680 g/kg	OP	III	–	–	–	2	0.7
	Faaba Soja 100 SL	Imazethapyr 100 g/l	Imidazolinones	III	–	–	1	–	0.3
Fungicide	Idefix WP	Cuprous oxide 65,5%	CU	II	–	–	–	1	0.3
	Compass 50 WG	Trifloxystrobin 50%	Pyr	U	–	1	–	–	0.3

^a^Pyr = Pyrethroid; Ave = Avermetin; OP = Organophosphate; Ary = Aryloxyacid; DTCs = Dithiocarbamates; CU = Composed of copper; T = Triazine derivative. ^b^ Ib = highly hazardous; II = moderately hazardous (WHO. 2005); III = Slightly hazardous; U = Unlikely to present acute hazard in normal use. ^c^ Ins = Insecticide; Aca = Acaricide; Her = Herbicide; Fon = fungicide

### Edible insect uses

**Fig. 7:**
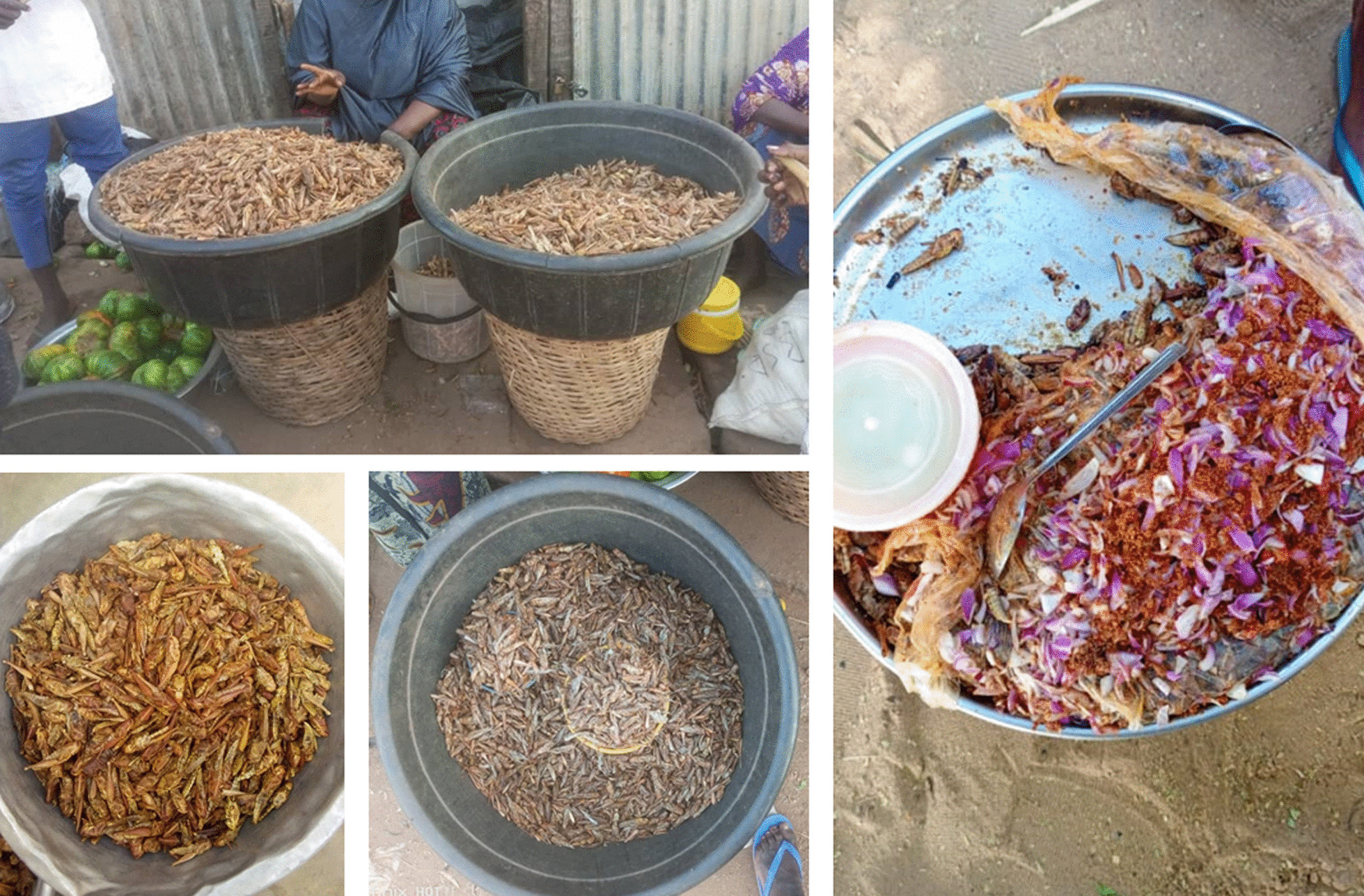
Some edible insect species sold in the Malanville markets

Besides their food use (63.2%), edible insects in the study area were used in traditional medicine (19%), spiritual uses (8.8%), poultry feed (6.1%), animal trap (1.3%), sold in markets (1%), and for fishing (0.6%). The use values of edible insects in the study area varied from 0.14 (*Cybister* sp.) to 1 (*B. membranaceus*) (Table 3). Eight edible insect species were used by the surveyed people of Atacora and Plateau departments to treat diverse ailments (Table 5). These insects are often mixed with honey for oral administration or shea butter for topical application. Among all the species of edible insects inventoried, fried grasshoppers (*A. ruficornis citrina*) and tobacco crickets (*B. membranaceus*) are the only ones listed as sold in markets mainly in the Alibori department (Fig. 7). These insects were sold either in piles or with a local measuring instrument called "Togolo" whose price varied depending on the insect and the period. Using the local "Togolo" measure, grasshoppers and crickets were sold at 1500FCFA and 2000 FCFA respectively in the dry season (November to March). While, in the rainy season (April to October), the price was falling ranging from 1000 to 1500FCFA for one "Togolo" of grasshoppers and crickets, respectively. In the Plateau department, *R. phoenicis* and *Spathosternum pygmaeum* Karsch were used by some people as bait to catch freshwater fish. While, *B. membranaceus* and *Gryllus bimaculatus* De Geer, *M. bellicosus*, *M. falciger*, *Ornithacris turbida cavroisi*, and *S. pygmaeum* were used for animal trap and poultry feed by some interviewed people of Atacora department. Except for *A. ruficornis citrina* and *Cybister* sp., the others listed as edible insects are used for spiritual purposes by the people interviewed mainly in the Atacora and Alibori departments.

**Table 5:** Edible insect species used in traditional folk medicine in the study area

Order	Family	Voucher number	Genera/ Species	Treated diseases	Used part	Preparation	Application	Use in combination with	Fidelity level (%)
Coleoptera	Dytiscidae	127	*Cybister* sp	Mumps	Whole insects	Powder	Auricular pathway	Black peppercorn	10
				Osteoarthritis	Whole insects	Dried and powdered	Topical	Shea Butter	10
	Dynastidae	142	*Oryctes ohausi* (Fabricius, 1793)	Yellow fever	Whole insects	Decoction	Oral	Water	10
	Scarabaeidae	144	*Gnathocera impressa* (Olivier, 1789)	Mumps	Head	Powder	Auricular pathway	Sea water	10
Hymenoptera	Formicidae	143	*Pheidole megacephala* (Fabricius 1793)	Fast walking for the disabled	Whole insects	Powder	Topical	Shea butter	5
Orthoptera	Gryllidae	134	*Brachytrupes membranaceus* (Drury, 1770)	Transformation of hoarse voice into fine voice	Whole insects	Fried	Oral	Onion	25
						Dried and powdered	Oral	Honey	
	Acrididae	138	*Hieroglyphus africanus* *(*Uvarov, 1922)	Anti-scorpion venom	Whole insects	powder	Topical	Shea butter	5
		139	*Acanthacris ruficornis citrina* (Serville, 1838)	Malaria	Whole insects	Dried and powdered	Oral	Fresh cow's milk	5
		137	*Ornithacris turbida cavroisi* (Finot, 1907)		Whole insects	Decoction	Oral	Water ginger	5
				Memory aid	Whole insects	Powder	Oral	Honey	5
				Scab	Thorax	Powder	Topical	Some plants	5
				Yellow fever	Whole insects	3 insects reduced to powder	Oral	Some plants	5

**Fig. 8:**
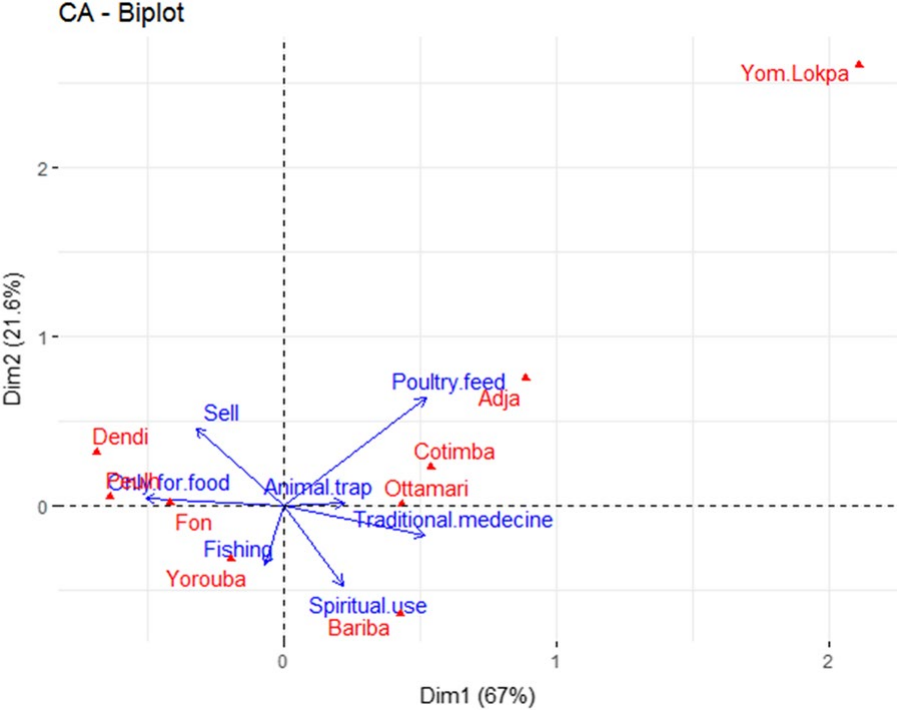
Results of Principal Components Analysis to describe the relationship between ethnic groups and traditional use of edible insect

The results of the principal component analysis showed that the factorial design formed by axis 1 and 2 explains 88.6% of the initial total variability of the different traditional uses of edible insects by ethnic groups (Fig. 8). The analysis showed that most surveyed people of Ottamari ethnic group used edible insects for traditional medicine and for animal trapping. While the surveyed people from the Bariba ethnic group mainly use edible insects for spiritual purposes. On the other hand, the surveyed people from the Adja and Cotimba ethnic groups mainly use edible insects to feed poultry. Respondents from the Dendi ethnic group are mainly involved in selling edible insects. While those of the Yoruba ethnic group use them for fishing. The majority of surveyed people of the Fon and Peulh ethnic groups used edible insects only for food.

### Perception of entomophagy

**Fig. 9:**
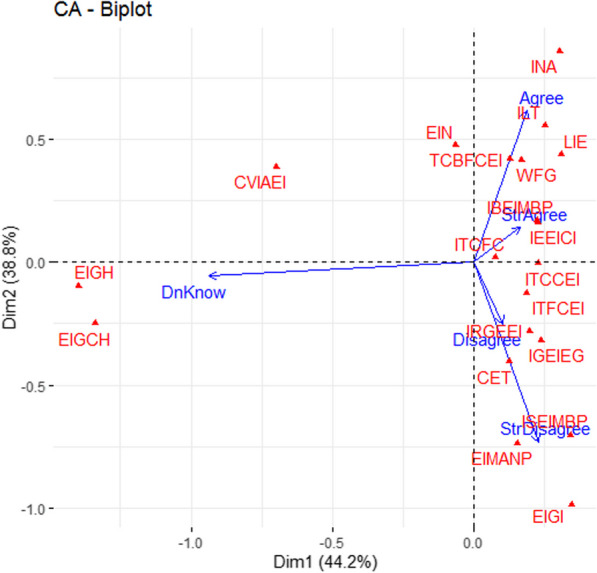
Results of Principal Components Analysis to describe respondents' perception of insect consumption. *ILE* I like to eat edible insects; *INA* I’m not afraid to eat edible insects or when I see them; *EIN* Edible insects are nutritious; *ILT* I like the taste of edible insects; *EIGH* Edible insects are good for the health; *WFG* When I eat edible insects, I feel good; *CET* The capture of edible insects is easy for me at any time in the year; *EIGCH* Edible insects are good for children’s health; *ITCFC* I teach my children and my friends to capture edible insects; *ITCCEI* I teach my children to consume edible insects; *ITFCEI* I teach my friends to consume edible; *IRGEEI* I recommend to my guests to eat edible insects; *IGEIEG* I give edible insects to eat to my guests; *IEEICI* I eat edible insects because it's part of my cultural identity; *EIGI* Edible insects generate me income; *TCBFCEI* Those who can afford to buy fish also consume edible insects; *EIMANP* Edible insects are more available now than in past years; *CVIAEI* Climatic variations greatly influence the availability of edible insects; *IBEIMBP* I will buy the edible insects when they are marketed with beautiful packaging; *ISEIMBP* I will sell edible insects when they will be marketed with beautiful packaging; *Disagree* Disagree; *DnKnow* Do not know; *StrAgree* Strongly Agree; *Agree* Agree; *StrDisagree* Strongly Disagree

The factorial design formed by the first two axes explains 70.3% of the initial total variability of the different perceptions of entomophagy made by the respondents (Fig. 9). The analyses revealed that the Linkert scale such as disagree and strongly disagree are positively correlated with axis 1 in contrast to those of agree or strongly agree. Axis 2 is negatively correlated with the neutral scale, don't know. In general, the overall pattern of different assessments towards entomophagy perception showed that respondents are not afraid to eat insects. The majority of respondents consider edible insects to be nutritious, and feel good after eating them and therefore, shows a willingness to purchase edible insects as a new manufactured product. More than half of the respondents consumed insects because of their delicious taste (85.6%) and 31.7% consumed them for their fish-like taste. Some respondents (40.3%) consumed insects because of the cultural values associated with them. However, the surveyed people do not recommend to guests or teach their friends to eat insects. It also appears from their response that edible insects are no longer available as before and they do not trade insects and therefore do not make them a source of income. Furthermore, they are unaware of the effect of insect consumption on the health and well-being of children, and also of the impact of climate change on the availability of insects.

**Fig. 10:**
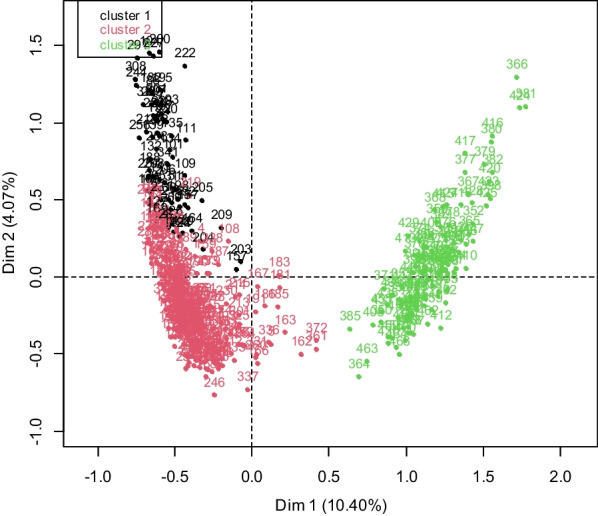
Projections of respondents in the scheme formed by the first two axes of the HCA. Cluster1: Insect consumers with a very negative perception of entomophagy; Cluster 2: Insect consumers with a positive perception of entomophagy; Cluster 3: Insect consumers with a very positive perception of entomophagy

The results of the Hierarchical Clustering on Principal Components (HCPC) showed that 79.52% of the initial variability of the perception measures and social characteristics was retained on the first 60 axes of the Multiple Correspondence Analysis (MCA). The hierarchical clustering analysis (HCA) identified three groups of perceptions related to sociocultural and economic characteristics (Fig. 10).

The first cluster, which included 14.31% of respondents was characterized by consumers with a very negative perception of entomophagy and are not ready to promote it (Table 6). These respondents are notably Ottamari (55.22%; *p* < 0.001) and Cotimba (13.43%; *p* = 0.004), animists (38.81%; *p* = 0.001) with a high level of education (secondary: 35.82%, *p* < 0.001; university: 8.96%, *p* = 0.001) and whose frequency of insect consumption is average (38.81%, *p* < 0.001). The second cluster included 59.40% of the respondents mainly Yorouba (36.69%, *p* < 0.001) and Ottamari (50.72%, *p* < 0.001) consumers with a positive perception of entomophagy and its promotion (Table 7). The majority of these respondents are adults (74.82%, *p* = 0.003) and older persons (11.87%, *p* < 0.001) who practice the religion of Christianity (57.91%, *p* < 0.001) and who have a high frequency of insect consumption (73.74%, *p* < 0.001). However, these respondents are unaware of the health benefits of eating edible insects and do not adhere to their sales. The third group including 26.29% of the respondents belonging to the Dendi (65.85%, *p* < 0.001) and Fon (21.95%, *p* < 0.001) ethnic groups, practicing Islam (73.17%, *p* < 0.001) who are very favourable to entomophagy and its promotion (Table 8). This acceptability of entomophagy is associated with a young age (32.52%; *p* < 0.001) and a low frequency of insect consumption (32.52%; *p* < 0.001). They have knowledge of the health benefits of edible insects and are willing to buy them despite their low monthly consumption.

**Table 6:** First grouping of perception variables according to socio-cultural and economic variables

Variables		Cla/Mod	Mod/Cla	*p*-Value	Vtest
Statements and associated socio-cultural and economic characteristics	Consumer perception				
I recommend to my guests to eat edible insects	Strongly disagree	81.33	91.04	< 0.001	15.69
I give edible insects to eat to my guests	Strongly disagree	72.09	92.54	< 0.001	15.02
I teach my friends to consume edible insects	Strongly disagree	90.57	71.64	< 0.001	13.91
I teach my children to consume edible insects	Strongly disagree	73.68	62.69	< 0.001	11.32
I teach my children and my friends to capture edible insects	Strongly disagree	74.47	52.24	< 0.001	10.14
When I eat edible insects, I feel good	Disagree	41.59	70.15	< 0.001	8.71
I will buy the edible insects when they are marketed with beautiful packaging	Strongly disagree	62.79	40.3	< 0.001	7.83
Edible insects generate me income	Strongly disagree	26.29	91.04	< 0.001	7.73
I eat edible insects because it's part of my cultural identity	Strongly disagree	56.86	43.28	< 0.001	7.69
I like the taste of edible insects	Disagree	45.95	50.75	< 0.001	7.39
Edible insects are good for children's health	Strongly disagree	59.09	38.81	< 0.001	7.38
I like to eat edible insects	Disagree	38.83	59.7	< 0.001	7.31
I will sell edible insects when they will be marketed with beautiful packaging	Strongly disagree	25	80.6	< 0.001	6.19
Those who can afford to buy fish also consume edible insects	Strongly disagree	47.5	28.36	< 0.001	5.3
I'm not afraid to eat edible insects or when I see them	Disagree	65	19.4	< 0.001	5.28
Edible insects are good for the health	Disagree	31.91	44.78	< 0.001	4.99
The capture of edible insects is easy for me at any time in the year	Strongly disagree	31.18	43.28	< 0.001	4.76
Edible insects are more available now than in past years	Strongly disagree	23.42	55.22	< 0.001	3.88
I like the taste of edible insects	Strongly disagree	80	5.97	< 0.001	3.12
Climatic variations greatly influence the availability of edible insects	Strongly disagree	57.14	5.97	0.01	2.56
Edible insects are nutritious	Disagree	30	13.43	0.02	2.28
Ethny group (Cotimba)		27.27	13.43	0.04	2.01
Ethnic group (Ottamari)		20.79	55.22	< 0.001	3.06
Highest level education (Secondary)		24.49	35.82	< 0.001	3.05
Religion (Animist)		22.22	38.81	0.01	2.7
Highest level education (University)		42.86	8.96	0.01	2.59
Monthly Consumption (Mean)		31.33	38.81	< 0.001	4.45

Clas/Mod = Proportion of respondents who specified this modality in this group compared to all respondents who specified this modality; Mod/Clas = Proportion of respondents who specified this modality in this group compared to all individuals in this group; *p*-value = Probability value

**Table 7:** Second grouping of perception variables according to socio-cultural and economic variables

Variables		Cla/Mod	Mod/Cla	*p*-Value	Vtest
Statements and associated socio-cultural and economic	Consumer perception				
I like the taste of edible insects	Agree	89.67	68.71	< 0.001	12.71
I teach my friends to consume edible	Disagree	92.64	54.32	< 0.001	11.42
Edible insects are good for children’s health	Do not know	87.5	62.95	< 0.001	11.1
Those who can afford to buy fish also consume edible insects	Agree	81.18	74.46	< 0.001	10.64
Edible insects are good for the health	Do not know	85.51	63.67	< 0.001	10.55
Edible insects generate me income	Disagree	100	28.78	< 0.001	9.45
I teach my children to consume edible insects	Disagree	88.97	43.53	< 0.001	8.79
Edible insects are more available now than in past years	Disagree	86.27	47.48	< 0.001	8.59
I recommend to my guests to eat edible insects	Agree	96.47	29.5	< 0.001	8.56
When I eat edible insects, I feel good	Agree	78.01	67.63	< 0.001	8.52
I eat edible insects because it's part of my cultural identity	Disagree	95.18	28.42	< 0.001	8.06
I give edible insects to eat to my guests	Agree	95.18	28.42	< 0.001	8.06
I teach my children and my friends to capture edible insects	Agree	85.14	45.32	< 0.001	8
The capture of edible insects is easy for me at any time in the year	Disagree	81.22	52.88	< 0.001	7.81
I teach my friends to consume edible insects	Agree	90.29	33.45	< 0.001	7.71
I eat edible insects because it's part of my cultural identity	Agree	81.25	51.44	< 0.001	7.65
I teach my children to consume edible insects	Agree	82.89	45.32	< 0.001	7.4
I will sell edible insects when they will be marketed with beautiful packaging	Strongly disagree	75	58.27	< 0.001	6.41
Climatic variations greatly influence the availability of edible insects	Do not know	80.47	37.05	< 0.001	5.85
I'm not afraid to eat edible insects or when I see them	Agree	65.9	82.01	< 0.001	4.76
The capture of edible insects is easy for me at any time in the year	Agree	74.68	21.22	< 0.001	3.07
I will buy the edible insects when they are marketed with beautiful packaging	Disagree	74.03	20.5	< 0.001	2.89
Edible insects are nutritious	Do not know	75.56	12.23	0.02	2.34
Age group (Adult)		62.65	74.82	0.03	2.22
Ethnic group (Ottamari)		79.21	50.72	< 0.001	6.97
Ethnic group (Yorouba)		78.46	36.69	< 0.001	5.32
Monthly Consumption (High)		66.78	73.74	< 0.001	4.45
Age group (Older person)		82.5	11.87	< 0.001	3.2
Religion (Christian)		74.54	57.91	< 0.001	6.21

Clas/Mod = Proportion of respondents who specified this modality in this group compared to all respondents who specified this modality; Mod/Clas = Proportion of respondents who specified this modality in this group compared to all individuals in this group; *p*-value = Probability value

**Table 8:** Third grouping of perception variables according to socio-cultural and economic variables

Variables		Cla/Mod	Mod/Cla	*p*-Value	Vtest
Statements and associated socio-cultural and economic characteristics	Consumer perception				
Edible insects generate me income	Strongly agree	91.3	68.29	< 0.001	15.2
I give edible insects to eat to my guests		88.54	69.11	< 0.001	14.92
I recommend to my guests to eat edible insects	Strongly agree	84.04	64.23	< 0.001	13.54
I eat edible insects because it's part of my cultural identity	Strongly agree	73.73	70.73	< 0.001	13.02
The capture of edible insects is easy for me at any time in the year	Strongly agree	95.16	47.97	< 0.001	12.58
Those who can afford to buy fish also consume edible insects	Strongly agree	80.46	56.91	< 0.001	12
I teach my children to consume edible insects	Strongly agree	81.18	56.1	< 0.001	11.98
Edible insects are more available than before	Strongly agree	90.63	47.15	< 0.001	11.85
I teach my friends to consume edible	Strongly agree	71.7	61.79	< 0.001	11.48
When I eat edible insects, I feel good	Strongly agree	85.29	47.15	< 0.001	11.2
The taste of edible insects pleases me	Strongly agree	62.07	58.54	< 0.001	9.64
Edible insects are good for the health	Strongly agree	90.91	32.52	< 0.001	9.53
I teach my children and my friends to capture edible insects	Disagree	96.97	26.02	< 0.001	8.99
Edible insects are good for children’s’ health	Strongly agree	78.57	35.77	< 0.001	8.77
Climatic variations greatly influence the availability of edible insects	Do not know	100	21.14	< 0.001	8.31
Edible insects are more available now than in past years	Disagree	100	16.26	< 0.001	7.17
I will sell edible insects when they will be marketed with beautiful packaging	Disagree	100	9.76	< 0.001	5.39
I'm not afraid to eat edible insects or when I see them	Strongly agree	46.81	35.77	< 0.001	4.83
Edible insects are nutritious	Strongly agree	35.58	60.16	< 0.001	4.06
I will buy the edible insects when they are marketed with beautiful packaging	Disagree	100	5.69	< 0.001	3.96
Edible insects are good for the health	Agree	50	21.95	< 0.001	3.95
I like to eat edible insects	Strongly agree	33.14	46.34	0.01	2.54
Religion (Muslim)		66.67	73.17	< 0.001	12.27
Age group (Young)		41.67	32.52	< 0.001	3.69
Monthly Consumption (Low)		51.28	32.52	< 0.001	5.19
Ethnic group (Fon)		81.82	21.95	< 0.001	6.88
Ethnic group (Dendi)		94.19	65.85	< 0.001	15.26

Clas/Mod = Proportion of respondents who specified this modality in this group compared to all respondents who specified this modality; Mod/Clas = Proportion of respondents who specified this modality in this group compared to all individuals in this group; *p*-value = Probability value

### Factors determining insect consumption

The test of chi-square or Fisher exact showed strong evidence (*p* < 0.001) that the proportion of participants who eat the insect is associated with the ethnic group, and religion and also linked to their accessibility of market (Table 9). On the other hand, there was no evidence that the consumption of the insect is associated with gender (*p* = 0.92), age group (*p* = 0.21), education (*p* = 0.54), and source of income (*p* = 0.98). The results of the full model taking into account all the factors indicated that the covariates considered in this model, with the exception of the ethnic group, religion, and market accessibility, were no evidence (*p* > 0.05) on edible insect consumption. Thus, this model leads to the same results as the univariate tests performed on each of the factors. Therefore, edible insect consumption is associated with an ethnic group, religion, and market accessibility. Indeed, more Ottamari respondents (*p* = 0.004) consume insects than other ethnic group participants (Table 10). And the Muslim respondents (*p* = 0.03) consume weakly insects than Christians and animists. As access to the market becomes increasingly difficult (*p* < 0.001), people consume more insects (Table 10).

**Table 9:** Factors influencing entomophagy practice in the study area

Variables	Levels	Respondents still practicing entomophagy (%)		*p*-Value
		No (N = 100)	Yes (N = 379)	
Gender	Female	38 (21.2)	141 (78.8)	0.92
	Male	62 (20.7)	238 (79.3)	
Age group	Young	26 (26.8)	71 (73.2)	0.21
	Adult	64 (18.8)	276 (81.2)	
	Older person	10 (23.8)	32 (76.2)	
Ethnic group	Adja	0 (0)	3 (100)	** < 0.001**
	Bariba	1 (33.3)	2 (66.7)	
	Cotimba	6 (18.2)	27 (81.8)	
	Dendi	33 (36.7)	57 (63.3)	
	Fon	4 (12.1)	29 (87.9)	
	Ottamari	16(8.84)	165(91.16)	
	Peulh	0 (0)	1 (100)	
	Yom-Lokpa	0 (0)	1 (100)	
	Yorouba	40 (29.9)	94(70.1)	
Religion	Animist	14 (11.9)	104 (88.2)	** < 0.001**
	Christian	39 (17.7)	181 (82.3)	
	Muslim	47 (33.3)	94 (66.7)	
Highest level of education	Illiterate	55 (20.5)	213 (79.5)	0.54
	Primary	18 (18.4)	80 (81.6)	
	Secondary	22 (22.5)	76 (77.5)	
	University	5 (33.3)	10 (66.7)	
Main sources of income for the family	Agriculture	49 (21.2)	182(78.8)	0.98
	Crafts	20 (19.8)	81 (80.2)	
	Employee	2 (25)	6 (75)	
	Trading	29 (21.2)	108 (78.8)	
	None	0 (0)	2 (100)	
Market accessibility	Easy	61 (57.5)	45(42.5)	** < 0.001**
	Difficult	37 (11.5)	284 (88.5)	
	Very difficult	2 (3.9)	49 (96.1)	
	No response	0 (0)	1 (100)	

*p*-value < 0.05% revealed a significant difference between the values of different levels of a variable and it was highlighted in bold

*p*-values asses the overall significance and are from the unadjusted test (Khi square or Fisher test); *n* number of respondents; % percentage

**Table 10:** Result of multivariable logistic regression analysis

Variable	Levels	Coef (Std Err)	OR	*p*-Value
Gender	Female	Reference	1	
	Male	− 0.22 (0.35)	0.8 [0.39, 1.60]	0.54
Age group	Adulte	Reference	1	
	Young	0.35 (0.38)	1.41 [0.67, 3.10]	0.36
	Older person	− 0.92 (0.52)	0.39 [0.15, 1.16]	0.07
Ethnic group	Yorouba	Reference	1	
	Ottamari	1.22 (0.42)	3.41 [1.51, 8.07]	0.004
	Cotimba	− 0.05 (0.66)	0.94 [0.26, 3.75]	0.93
	Others	0.60 (0.43)	1.82 [0.79, 4.32]	0.16
Religion	Animist	Reference	1	
	Christian	− 0.25 (0.46)	0.78 [0.30, 1.90]	0.58
	Islam	− 1.07 (0.51)	0.34 [0.12, 0.91]	0.03
Highest level of education	Illiterate	Reference	1	
	Primary	0.23 (0.41)	1.27 [0.57, 2.94]	0.56
	Secondary	− 0.51 (0.43)	0.60 [0.26, 1.42]	0.24
	University	− 0.55 (0.80)	0.57 [0.12, 2.92]	0.49
Main sources of income for the family	Agriculture	Reference	1	
	Crafts	0.13 (0.41)	1.14 [0.51, 2.62]	0.75
	Employee	1.12 (1.10)	3.07 [0.39, 35.57]	0.31
	None	10.96 (698.97)	57 [ − 13, 0]	0.99
	Trade	0.08 (0.39)	1.09 [0.51, 2.38]	0.83
Market accessibility	Easy	Reference	1	
	Difficult	2.42 (0.33)	1.12 [5.99, 22.05]	< 0.001
	Very difficult	3.31 (0.87)	2.75 [6.36, 32.92]	< 0.001

*Std Err* Standard error; *OR* Odd-ratio

## Discussion

Our results show that the consumption of insects goes back a long time and more frequent in the Atacora region than in other prospected regions. In fact, the population of this region is more affected by food insecurity [31], relies more heavily on entomophagy to meet their nutritional needs [12]. However, a small proportion (10–30%) of edible insects contribute to the nutrient’s intake of the surveyed populations with low-frequency consumption. This is not surprising, because some studies showed that insects are the least accepted alternative sources of nutrients among the population [32, 33]. Nevertheless, it would be interesting to assess the nutritional impact of edible insects in the function of their frequency consumption in Beninese households. In addition, knowing that the form of consumption can affect the digestibility and the nutritional properties of edible insects, therefore, it is important to check whether the fried form preferred by the Beninese populations allows optimal assimilation of nutrients.

A difference in the frequency of insect consumption was observed with regard to the level of education, age, and religion of the respondents. Indeed, it is known that certain religious prohibitions can slow down entomophagy [4], and that education can lead to the abandonment of the consumption of insects [34]. In addition, several studies have shown that young people are more inclined to entomophagy than older people mainly because of their openness [35, 36] Like Zimbabweans [37] and Ivorians [38], the delicious taste of insects is one of the main reasons for their consumption. In the study area, the cultural values associated with certain species of insects also contribute to the perpetuation of their consumption from generation to generation. Indeed, Hlongwane et al. [4] and Ghosh et al. [11] showed that culture plays an important role in entomophagy acceptance and edible insect preference.

Our results showed that 17 species of insects are consumed in the four surveyed regions. This number of edible insect species is low comparatively to other studies conducted in Benin [14, 15], and could reflect a gradual disappearance of some species or the decreasing trend of insect availability. Among the recorded species, *Lampetis gorilla* (Thomson) (Coleoptera: Buprestidae) to our knowledge has never been mentioned as edible insect in Benin. This could suggest that entomophagy is experiencing a positive development in Benin with new species of insects consumed. The dominance of Orthoptera, Blattaria, and Coleoptera among the edible insects in Benin is not surprising. Indeed, these insects’ orders are in the top five of most commonly consumed in Sub-Saharan Africa [39], and across the world [40, 41]. As mentioned by Ebenebe et al. [42], in Nigeria, the Tobacco cricket *B. membranaceus* is among the most preferred edible insects.

The use of chemical pesticides to protect farmlands was the main constraint hindering the accessibility of edible insects to populations. Indeed, edible insects are mainly caught in cropping areas, where they are also considered by some farmers as crop pests and killed with pesticides. In fact, some studies showed that edible insects collected in crop fields have a high quantity of pesticide residues on and recommended their farm rearing according to appropriate regulations to ensure safety [43, 44]. It would therefore be interesting to assess the level of pesticide residues contained in the main edible insects consumed in Benin. It is therefore urgent to develop simple methods of producing edible insects, accessible to low-income populations in order to guarantee the security of populations and contribute to poverty reduction.

Diverse uses of edible insects were recorded in the study area. As in other countries, edible insects are used for medicinal purposes [45–47], cultural rituals [48], and for poultry feed [49]. The market dedicated to edible insects is very little developed in Benin and remains informal. Indeed, Riggi et al. [14] mentioned the collection and informal sale of edible insects by the Nagot in southern Benin towards Nigeria. The influence of market accessibility on edible insect frequency consumption is not surprising. Indeed, respondents who have difficult access to the market consumed the traditional local products available to them to meet their nutritional needs and consider edible insects which are also of the same quality than the ‘meat’ from traditional sources [50] as their main food source. The grasshopper species (*A. ruficornis citrina* and *B. membranaceus*) mentioned as sold in the markets of Alibori department were also cited as having a commercial value in Nigeria [42], and Cameroon [51]. It is therefore appropriate to create a value chain around edible insects and thus contribute to the establishment of a formal market.

The perception of entomophagy and the frequency of consumption of edible insects depends on ethnic groups. In fact, Dendi, Fon, Yorouba and Ottamari ethnic groups showed a good perception of entomophagy and clear desire to promote it. However, some respondents of Yorouba and Ottamari ethnic groups are unaware of the health benefits of eating edible insects. This ignorance of the nutritional benefits of edible insects despite their consumption could be explained by the fact that they consume the insects only by food habit or just for flavour. It is, therefore, necessary to organize awareness-raising on the nutritional benefits linked to the consumption of edible insects towards this type of consumer to reinforce their inclusion as a regular source of nutrients in their diet.

Our results showed that respondents from the Cotimba and Ottamari ethnic groups, who still practice endogenous religions have a negative perception of entomophagy compared to those who are Christians or Muslims. Indeed, it is known that religions prohibiting the consumption of animals and their derivatives significantly influence the practice of entomophagy [52, 53]. It is therefore not surprising that religion was found to be an important factor influencing consumption frequency in the study area. In addition, eating insects is not prohibited for Christians and Muslims because it is mentioned in various literature related to these religions [54, 55]. However, knowing that the cultural realities [56] and some religions such as the Zionist churches in South Africa [57] or the apostolic churches in Zimbabwe [37] can negatively influence insect consumption, it is important to evaluate the effect of religious and cultural beliefs on entomophagy practice in Benin in order to define appropriate policies for its promotion.

## Conclusion

Our results showed that the consumption of edible insects represents a small part of the total food intake, and is however fully rooted in the eating habits of the surveyed people. Several reasons motivating the consumption of insects have been recorded and must be exploited in the definition of policy to promote entomophagy in the study area. Various synthetic chemical pesticides are used in edible insect collection fields suggesting the possibility of consuming insects containing residues harmful to the health of populations. The development and popularization of edible insect farming methods accessible to low-income populations is therefore recommended. In addition, awareness sessions on the nutritional benefits associated with the consumption of edible insects must be organized for their regular inclusion in the diet of populations. A differential perception of entomophagy across ethnic groups was noted with a negative perception among the Cotimba ethnic group. Ethnicity and religion are the main factors taken into account in the definition of policies to promote entomophagy in Benin.

## Data Availability

Raw and treated data generated during study are available on the link https://doi.org/10.6084/m9.figshare.24151566
